# Combatting PDAC with two tumor-targeting small RNAs

**DOI:** 10.18632/oncotarget.27245

**Published:** 2019-10-15

**Authors:** Andrea L. Kasinski

**Affiliations:** Department of Biological Sciences, Purdue University, West Lafayette, IN 47907, USA; Purdue Center for Cancer Research, Purdue University, West Lafayette, IN 47907, USA

**Keywords:** PDAC, microRNA, miR-217-5p, KRAS, siRNA

***News on***: *Tumor penetrating nanomedicine targeting both an oncomiR and an oncogene in pancreatic cancer by Gilles et al. Oncotarget. 2019; 10:5349–5358. https://doi.org/10.18632/oncotarget.27160*

Modulating the expression of protein-coding genes and microRNA-coding genes that are essential for maintaining a particular disease state has the potential to dramatically alter the trajectory of the disease. For example, the oncoprotein KRAS and the oncomiR microRNA-21 (miR-21) are important drivers of multiple cancers, including pancreatic ductal adenocarcinoma (PDAC). Thus, reducing the levels of KRAS and sequestering miR-21 at the same time could represent a powerful approach to targeting PDAC. This was the hypothesis tested by Slack and colleagues.

PDAC originates from the acinar cells of the exocrine pancreas. Early stages of disease are marked by reprogramming of the acinar cells towards a ductal phenotype driven by an early molecular change in the KRAS oncoprotein, resulting in constitutive pro-growth signaling [1]. Following additional genetic insults, including mutations in the tumor suppressor p53, early pancreatic intraepithelial neoplasias (PanINs) differentiate into invasive PDAC. When PDAC has develop and becomes invasive, patients tend to be refractory to treatment, with current treatment regiments only extending survival by ~10%, making PDAC the second leading cause of cancer-related deaths. In addition to the contribution of canonical protein-coding gene alterations, such as KRAS and TP53 to PDAC, miRNA-coding genes also contribute to disease progression and treatment response. For example, one of the most commonly upregulated oncogenic miRNAs, miR-21 is elevated in PDAC and miR-21 sequestration can reduce the growth of PDAC tumors highlighting the importance of this miRNA to the tumor cells [2]. Indeed, miRNA sequestration and miRNA overexpression therapeutics are making their way towards the clinic [3–5].

Targeted therapeutic approaches are typically favored over broad-spectrum agents due to their reduced toxicity. However, targeted agents faulter in that tumor cells can easily become resistant to these very specific agents. Resistance usually occurs due to the use of molecular bypass tracks where cells are no longer dependent on the targeted signaling pathway, or through acquired secondary mutations that reduce the efficacy of the drug. To circumvent these potential issues, a combinatorial targeted therapeutics approach is favored. In the present study published in this issue of Oncotarget, Slack and colleagues report on the use of a novel dual-targeted therapeutic agent that was developed based on the genetics of PDAC. In this case, focusing on the dependency of PDACs to two insults, oncogenic KRAS signaling and elevated levels of miR-21. Because most mutations of KRAS are considered undruggable, to specifically target KRAS, the authors attempted to downregulate KRAS using two separate RNA-targeting approaches. One approach made use of a miRNA that is often downregulated in PDAC that directly modulates KRAS expression, miR-217-5p [6]. The other used a more direct, and perhaps more potent approach through targeting KRAS with an siRNA.

Firstly, the team tested the combination of miR-217-5p, which was previously identified as a direct repressor of KRAS [6] and was found downregulated in PDAC samples, and an antimiR generated to sequester miR-21. Initially, there was no combinatorial benefit observed when miR-217-5p was co-delivered with antimiR-21; however, this observation guided the investigators on a path to change the method of downregulating KRAS to a more robust approach. They reasoned that perhaps a more specific RNA, directly targeting KRAS, combined with antimiR-21 would produce the hypothesized combinatorial response. Using the more aggressive siRNA-mediated KRAS targeting approach the team ultimately determined that downregulating KRAS while simultaneously reducing miR-21 could in fact further reduce the metabolic activity of the tumor cells, and increase apoptotic cell death and tumor regression in vivo over each RNA used independently. For the in vivo studies, both RNAs were delivered using a using tumor penetration nanocomplex, which can penetrate the dense architecture of the PDAC tumor stroma [2]

Without question the future of medicine and therapeutics will capitalize on both a targeted approach and a combinatorial approach. With the number of agents and targets available, the difficulty is in selecting the right target(s) and using the correct agent(s) that can perturb the target to the point of producing a therapeutic benefit. These sorts of challenges come with trial and error. The work by Slack and colleagues highlights this and begins to define a potential pipeline for identifying the correct combinatorial targeting regiment. Firstly, based on the molecular genetics of the disease, it is critical to identify targets that when impaired will have a direct consequence for the disease, as was the case for altering KRAS and miR-21 in PDAC. Secondarily, an arduous effort needs to be placed on assessing, and reassessing various mechanisms for targeting the intended target – in this case, not all miRNA- or siRNA- targeting approaches were equivalent. Thirdly, specific delivery of the small RNAs to the intended tissues in vivo is critical to avoid off-targeting and to potentially reduce dosing [7, 8]. Finally, as is the case with most targeted therapeutic agents, and was an observation made in the current study, not all tumors respond equivalently to the same agents. Some tumors continued to progress following treatment while others, in this case three, regressed. The reason for this variable response is currently unclear but is under intense investigation and likely depends on either genetics or the variability of the model system in use. For future clinical advancement of such agents, the reasons behind the responders and non-responders will need to be further evaluated. Nonetheless, this work underscores the importance of selecting the correct targets and the correct targeting agents for the design of robust combinatorial therapeutics.

## References

[1] Morris JP, et al Nature Reviews Cancer. 2010; 10:683–695. 10.1038/nrc2899. 20814421 PMC4085546

[2] Gilles ME, et al Clin Cancer Res. 2018; 24:1734–1747. 10.1158/1078-0432.CCR-17-2733. 29330203

[3] Kasinski AL, et al Nature Reviews Cancer. 2011; 11:849–864. 10.1038/nrc3166. 22113163 PMC4314215

[4] Orellana EA, et al Cancers (Basel). 2015; 7:1388–1405. 26226002 10.3390/cancers7030842 PMC4586775

[5] Kasinski AL, et al Curr Opin Mol Ther. 2010; 12:147–157. 20373258

[6] Zhao WG, et al Carcinogenesis. 2010; 31:1726–1733 (2010). 10.1093/carcin/bgq160. 20675343

[7] Orellana EA, et al Science Translational Medicine. 2017; 9:eaam9327. 10.1126/scitranslmed.aam9327. 28768807 PMC5576188

[8] Myoung S, et al Royal Society of Chemistry, Cambridge. 2019; 386–415. 10.1039/9781788016421-00386.

